# Precision Prediction for Dengue Fever in Singapore: A Machine Learning Approach Incorporating Meteorological Data

**DOI:** 10.3390/tropicalmed9040072

**Published:** 2024-03-29

**Authors:** Na Tian, Jin-Xin Zheng, Lan-Hua Li, Jing-Bo Xue, Shang Xia, Shan Lv, Xiao-Nong Zhou

**Affiliations:** 1 National Institute of Parasitic Diseases, Chinese Center for Disease Control and Prevention (Chinese Center for Tropical Diseases Research), Shanghai 200025, China; tina_t123@163.com (N.T.); xuejb@nipd.chinacdc.cn (J.-B.X.); sxia@nipd.chinacdc.cn (S.X.); lvshan@nipd.chinacdc.cn (S.L.); 2 School of Public Health, Shandong Second Medical University, Weifang 261000, China; orchid8@sina.com; 3 School of Global Health, Chinese Center for Tropical Diseases Research, Shanghai Jiao Tong University School of Medicine, Shanghai 200025, China; jamesjin63@163.com

**Keywords:** prediction, dengue fever, machine learning, meteorological data

## Abstract

Objective: This study aimed to improve dengue fever predictions in Singapore using a machine learning model that incorporates meteorological data, addressing the current methodological limitations by examining the intricate relationships between weather changes and dengue transmission. Method: Using weekly dengue case and meteorological data from 2012 to 2022, the data was preprocessed and analyzed using various machine learning algorithms, including General Linear Model (GLM), Support Vector Machine (SVM), Gradient Boosting Machine (GBM), Decision Tree (DT), Random Forest (RF), and eXtreme Gradient Boosting (XGBoost) algorithms. Performance metrics such as Mean Absolute Error (MAE), Root Mean Square Error (RMSE), and R-squared ($R^{2}$) were employed. Results: From 2012 to 2022, there was a total of 164,333 cases of dengue fever. Singapore witnessed a fluctuating number of dengue cases, peaking notably in 2020 and revealing a strong seasonality between March and July. An analysis of meteorological data points highlighted connections between certain climate variables and dengue fever outbreaks. The correlation analyses suggested significant associations between dengue cases and specific weather factors such as solar radiation, solar energy, and UV index. For disease predictions, the XGBoost model showed the best performance with an MAE = 89.12, RMSE = 156.07, and $R^{2}$ = 0.83, identifying time as the primary factor, while 19 key predictors showed non-linear associations with dengue transmission. This underscores the significant role of environmental conditions, including cloud cover and rainfall, in dengue propagation. Conclusion: In the last decade, meteorological factors have significantly influenced dengue transmission in Singapore. This research, using the XGBoost model, highlights the key predictors like time and cloud cover in understanding dengue’s complex dynamics. By employing advanced algorithms, our study offers insights into dengue predictive models and the importance of careful model selection. These results can inform public health strategies, aiming to improve dengue control in Singapore and comparable regions.

## 1. Introduction

Dengue fever (DF) is an acute infectious disease caused by dengue viruses, and is mainly transmitted by *Aedes* mosquitoes [1]. The virus has five distinct types [2], and infection with one type does not provide long-term immunity against the other types [3]. There is no specific treatment for dengue fever, and while a vaccine is available, its use is not universally recommended due to varying efficacy rates among different populations and the potential for severe outcomes if given to individuals who have never been infected with dengue [4]. According to WHO reports, the disease is currently prevalent in more than 100 countries in Africa, the Americas, the Eastern Mediterranean, Southeast Asia, and the Western Pacific, with the Americas, Southeast Asia, and the Western Pacific being the most severely affected [1]. It is predicted that by 2085, DF will have a wider impact, posing a threat for 50% to 60% of the global human population [5], and will become a significant public health concern [6].

In the tropical city-state of Singapore, despite ongoing rigorous vector control measures and public health interventions, DF continues to be a persistent issue. The existing measures to combat dengue, including vector control, public education, and vaccination (CYD-TDV, Dengvaxia), have shown limitations [7], highlighting the need for innovative and effective strategies. Therefore, there is a pressing need to develop more precise and robust prediction models for DF. Presently, Singapore ought to embark upon research endeavors aimed at constructing more precise and robust prediction models that can anticipate dengue risk and potential outbreak areas.

While the relationship between meteorological factors and the transmission dynamics of DF is well recognized [8], the existing models often utilize traditional statistical methods that may not fully capture the complex, non-linear relationships between these variables. Traditional epidemiological models often rely on assumptions that do not sufficiently account for the intricacies of dengue transmission, thus limiting the accuracy of their predictions. Traditional epidemiology tends to concentrate on identifying individual risk factors, which can be challenging for elucidating the complete etiological network and may present significant limitations when studying complex diseases. This approach imposes constraints on the types of data that can be effectively utilized for etiological inference [9]. In contrast, machine learning offers a promising avenue for addressing nonlinearity and interactions among variables [10], while also adeptly handling multi-dimensional datasets. Furthermore, the current prediction models for DF primarily rely on historical dengue case data. These models might not fully capture the impact of fluctuations in meteorological conditions on dengue transmission. Consequently, they may fail to predict unexpected outbreaks driven by changes in weather patterns. Many existing models do not sufficiently account for the time-lagged effects of weather variables on dengue transmission. The lifecycle of the *Aedes aegypti* mosquito and the incubation period of the dengue virus mean that changes in weather can have effects that are not immediately apparent [11]. Models that do not take these lags into account might miss crucial aspects of dengue transmission dynamics.

Given these limitations, there is a clear need for a more comprehensive, data-driven approach to predict the risk of DF in Singapore. Machine learning models have the potential to uncover complex, non-linear relationships and patterns within data that are not readily apparent using traditional statistical methods [12,13]. The proposed study aims to address these gaps by developing a precision model for risk prediction based on machine learning algorithms using meteorological data. The integration of diverse meteorological datasets into machine learning frameworks, coupled with algorithm selection for optimal model performance, has the potential to enhance the accuracy and timeliness of dengue fever (DF) predictions. This could allow for more effective and proactive public health interventions, ultimately contributing to the mitigation of dengue fever’s public health burden in Singapore.

## 2. Method

Data collection. The weekly DF case data in Singapore from 2012 to 2022 came from the public data website (https://data.gov.sg/) (accessed on 15 January 2023). The meteorological data of Singapore’s weekly temperature, humidity, rainfall, wind speed, sea level pressure, solar radiation, and other factors from 2012 to 2022 were from the weather data service website (https://www.visualcrossing.com/) (accessed on 16 January 2023). The following describes our approach in handling the week time-series of 583 datasets, with each dataset representing a unique combination of meteorological parameters and the corresponding dengue incidence data.

Data processing. Each dataset was thoroughly inspected for missing, inconsistent, and anomalous data. Any missing values were imputed using appropriate techniques with mean imputation or multiple imputations depending on the nature and extent of the missing data. All datasets were standardized to ensure consistency and comparability across different measurement scales.

Feature Engineering. Feature engineering was carried out to create meaningful variables that could potentially enhance the model’s predictive performance. This includes creating lag variables to account for the delayed impact of meteorological conditions on dengue transmission.

Data Splitting. The datasets were randomly split into a training set (80%) and a testing set (20%). The training set was used to build the machine learning models, while the testing set was reserved for final model validation to assess how well the model generalizes to unseen data.

Model Development. Various machine learning algorithms were employed, including General Linear Model (GLM), Support Vector Machine (SVM), Gradient Boosting Machine (GBM), Decision Tree (DT), Random Forest (RF), and eXtreme Gradient Boosting (XGBoost) algorithms. For each algorithm, a range of model parameters was tested using a grid search approach coupled with cross-validation on the training data to identify the most effective model configuration.

Model Evaluation and Selection. Each model’s performance was evaluated using the appropriate metrics: Mean Absolute Error (MAE), Root Mean Square Error (RMSE), and R-squared ($R^{2}$). The models were compared based on these metrics, and the best performing model was selected for further validation.

Model Validation. The final step involved validating the selected model using the testing set. This step provides an unbiased assessment of how the model is likely to perform in real-world scenarios, as the testing set represents unseen data.

The flowchart of the machine learning method is shown in Supplementary Figure S1.

Statistics. The analysis and model development in this study were performed using the open-source software R (version 4.0.3), renowned for its robustness in statistical computing and graphics. Several packages were used for the different stages of data preprocessing, analysis, and model development. The ‘tidyverse’ package facilitated data manipulation and visualization. ‘mice’ was used for handling missing data, while ‘caret’ facilitated data partitioning, model training, and performance assessment. The machine learning-specific packages ‘glmnet’, ‘e1071’, ‘gbm’, ‘rpart’, ‘randomForest’, and ‘xgboost’ were employed for implementing the General Linear Model (GLM), Support Vector Machine (SVM), Gradient Boosting Machine (GBM), Decision Tree (DT), Random Forest (RF), and eXtreme Gradient Boosting (XGBoost) algorithms, respectively.

Ethical Approval. Given the nature of this study, which relies exclusively on the secondary analysis of anonymized meteorological and public health data, ethical approval was not required. The datasets used were collected and made available by public health agencies and meteorological departments and did not contain any identifiable personal information. 

## 3. Results

### 3.1. Descriptive Analysis 

#### 3.1.1. Epidemic of Dengue in Singapore

Within the temporal scope of 2012 to 2022, there was a total of 164,333 cases of dengue fever. The incidence of DF in Singapore exhibited significant fluctuations with discernible epidemic trends. Notably, an ascending trend was observed from 2012 to 2013, followed by a declining trajectory from 2013 to 2018. This was succeeded by a resurgence in cases from 2018 to 2020, culminating in a zenith of incidence in 2020, which denotes the most substantial outbreak in recent years. A contraction was observed in 2021, succeeded by a resurgence in DF cases in 2022. Over the duration of the previous eleven years, a total of 164,333 instances of DF was recorded. The annual incidence of DF cases manifested considerable variability, with the year 2017 recording the minimum number of cases at 2767. Conversely, the year 2020 marked a peak in this epidemiological trend, witnessing the maximum case count of 35,315. 

**Figure 1:**
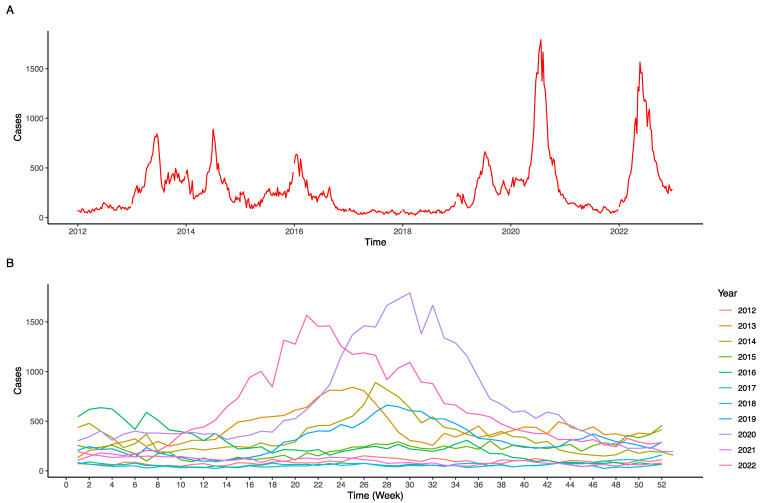
Trends of DF infections in Singapore from 2012 to 2022. (**A**) Annual number of DF cases in Singapore from 2012 to 2022. (**B**) Weekly number of DF cases in Singapore from 2012 to 2022.

Examination of the weekly incidence data for DF reveals a distinct seasonal pattern in its prevalence. Commencing from the 10th week of the year, an escalating trend was evident, aligning with the transition from March to July. The incidence apex was discernible between May and mid-July, encompassing the 20th to 30th weeks. Subsequent to the 30th week, a gradual decrement in case counts was observed, indicating a decline in DF prevalence (Figure 1A,B).

Over the course of the study period, the case fatality rate (CFR) associated with DF demonstrated variability. The year 2018 recorded the highest CFR at 1.52 per thousand cases, underlining the severity of that year’s outbreak. In contrast, the year 2017 witnessed an optimal scenario with a CFR of zero, signifying no mortality attributed to DF for that year. From 2012 to 2022, there was a statistically significant variance in dengue CFR (χ² = 32.62, *p* < 0.001) (Supplementary Table S1).

#### 3.1.2. Temporal Sequence of Climatological Variables

Statistical computations were executed on a dataset comprising 583 distinct meteorological observations, with key metrics such as mean, median, and standard deviation calculated for a set of 19 climatological variables including Tempmax, Tempmin, Temp, and Feelslikemax. The derived statistics are tabulated in Supplementary Table S2. Supplementary Figure S2 shows the chronological trajectories of these meteorological variables.

#### 3.1.3. Correlation Analysis among Variables

**Figure 2:**
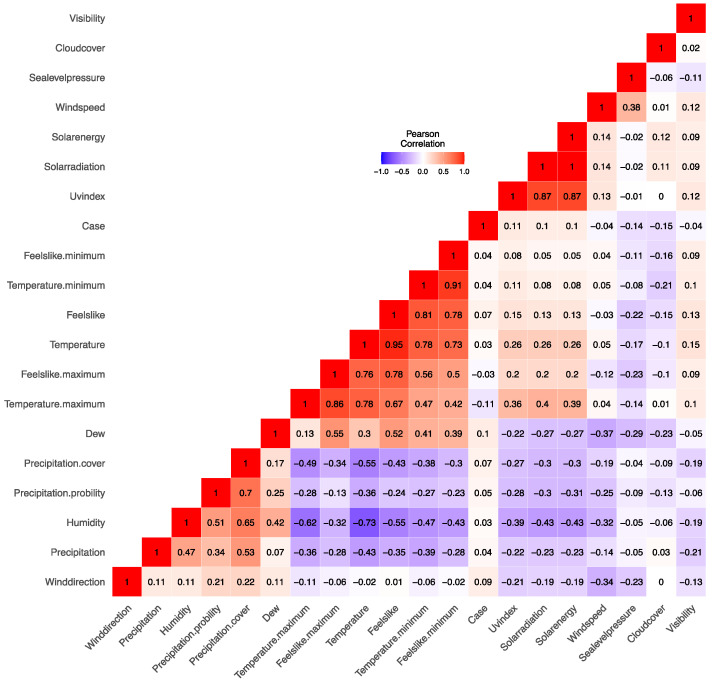
Spearman’s correlation matrix of continuous variables.

We investigate the associations among our predictor variables, employing Spearman’s rank correlation coefficient to quantify the statistical interdependencies. Figure 2 presents the correlation matrix delineating the relationships between the continuous predictors within our dataset.

An examination of the correlation coefficients among the variables presented in Supplementary Table S3 reveals that the variables Solarradiation, Solarenergy, and Uvindex exhibited substantial correlation coefficients (0.22, 0.22, 0.21, greater than 0.1) with DF cases. The relatively low magnitudes of these coefficients may imply that the prevalence of DF is potentially associated with certain delayed meteorological factors. 

### 3.2. Machine Learning Prediction of Dengue in Singapore

Considering the delayed influence of meteorological determinants on DF transmission and the cyclical periodicity inherent to the disease’s propagation, we integrated lagged values ranging from 1 to 12 weeks for each of the 19 meteorological parameters, culminating in the formulation of four distinct modes.

Mode 1. Incorporating both lag effects and temporal factors;
Mode 2. Considering only the lag effects;
Mode 3. Focusing solely on temporal factors;
Mode 4. Neglecting both lag effects and temporal factors.

### 3.3. Evaluation of the Efficacy of Various Predictive Models

The predictive efficacy of six distinct models was assessed in relation to their capacity to forecast DF cases. The specific performance metrics for each model of Modes 1–4 are delineated in Supplementary Tables S4–S7. As shown in Table 1, based on the model evaluation metrics, the best-performing model among the four modes was the XGBoost model in Mode 1, with an MAE of 89.12, RMSE of 156.07, and $R^{2}$ of 0.83. In the other three modes, the highest $R^{2}$ observed was only 0.50; Supplementary Figure S3 presents a scatter plot of Mode 1 illustrating the observed versus predicted dengue cases in Singapore utilizing the GLM, SVM, GBM, DT, RF, and XGBoost models. Upon examination of the plots and associated metrics, it can be inferred that the XGBoost model outperformed the remaining five models in Modes 1, 3, and 4, while in Mode 2, the SVM model performed the best. In Mode 1, the predictions produced by the XGBoost model closely aligned with the observed cases, as depicted in Supplementary Figure S3. The figures corresponding to Modes 2, 3, and 4 can be found in Supplementary Figures S4, S5 and S6, respectively.

### 3.4. Model Interpretation

We conducted an in-depth analysis of the most optimal model, XGBoost, in Mode 1, Table 2. The relative significance of each variable was normalized, with a maximum value of 1, where elevated scores denote a greater variable influence. Within this model, we identified the top 10 salient variables, with Week (0.54), Cloudcoverlag1 (0.10), Cloudcoverlag5 (0.07), Preciplag5 (0.03), Cloudcover (0.02), Dewlag7 (0.02), Tempmax (0.02), Cloudcoverlag7 (0.01), Cloudcoverlag3 (0.01), and Dewlag3 (0.01) being the foremost contributors.

**Figure 3:**
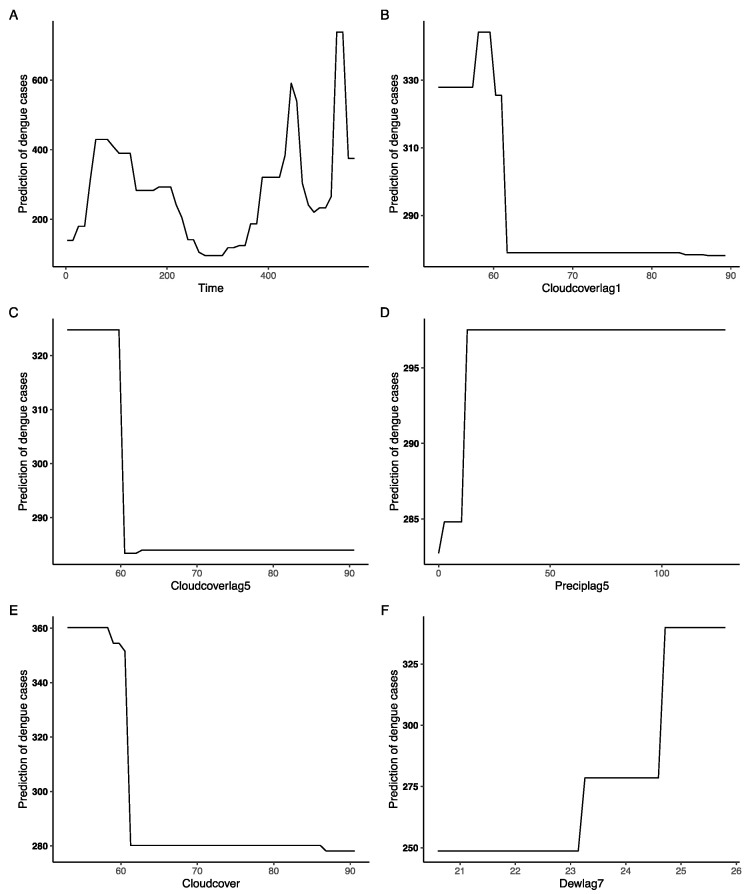
Dependence plots of the top six variables based on XGBoost model in Mode 1. (**A**) Dependence plot of the week and cases based on XGBoost model in Mode 1. (**B**) Dependence plot of the Cloudcoverlag1 and cases based on XGBoost model in Mode 1. (**C**) Dependence plot of the Cloudcoverlag5 and cases based on XGBoost model in Mode 1. (**D**) Dependence plot of the Preciplag5 and cases based on XGBoost model in Mode 1. (**E**) Dependence plot of the Cloudcover and cases based on XGBoost model in Mode 1. (**F**) Dependence plot of the Dewlag7 and cases based on XGBoost model in Mode 1.

Interdependencies between predictors and dengue incidences. Based on the variable significance scores extracted from the XGBoost algorithm, the top 18 predictors exhibited associations with dengue incidences (refer to Supplementary Figure S7). However, the magnitude and nature of these associations varied, with each displaying a non-linear correlation (Figure 3). 

The epidemiology of DF exhibits cyclical oscillations. A surge in cases was noted from 2012 to 2013, succeeded by a descending trend from 2013 to 2018. Thereafter, an uptick in incidences was observed from 2018 to 2020, reaching a peak in 2020, signifying the most pronounced outbreak in contemporary years. A decrease was documented in 2021, subsequently followed by a re-escalation of DF cases in 2022. When the cloud cover lag values at the first and fifth weeks, namely Cloudcoverlag1 and Cloudcoverlag5, exceeded 60, a pronounced reduction in DF cases was observed, which was maintained at a subdued level. This suggests a potential association between DF incidence and the cloud cover rate and its delayed effects. When the fifth-week lag value for precipitation, Preciplag5, exceeded 10, there was a sharp incline in the number of DF cases, which then persisted at elevated levels, implying that the DF incidence might be correlated with the rain volume with a delay of five weeks. The DF incidences exhibited a stepwise elevation in correlation with Dewlag7, initiating an increase at 23.2 and peaking at 24.7, indicating that DF incidence might be associated with the dew point temperature with a lag of seven weeks.

## 4. Discussion

From 2012 to 2022, Singapore’s DF incidence showed varied epidemic trends, with notable peaks in 2013 and 2020, the latter being the most significant outbreak with 35,315 reported cases [14]. In 2013, Singapore used the gravitrap for *Aedes* sentinel surveillance [15], and in 2016, the country carried out a phased testing approach of releasing Wolbachia-infected male *Aedes* mosquitoes to control dengue in Tampines, Yishun, and Braddell Heights [16], and thus a declining trajectory from 2013 to 2018 was observed. The largest documented dengue outbreak in 2020, consisting of 31,315 reported cases, occurred simultaneously with the implementation of COVID-19 control measures [17]. Singapore’s consistently warm and humid climate creates ideal circumstances for the proliferation and persistence of *Aedes aegypti* mosquitoes. There exists a robust seasonal pattern in the temporal distribution of dengue infections, characterized by a peak occurring typically in the vicinity of the mid-year period. The weekly incidence also showcased a palpable seasonality, amplifying during the transition from March to July, reminiscent of earlier findings linking meteorological variations to dengue transmission [8]. Detailed visual representations of these trends are shown in Supplementary Table S1 and Figure 1.

In the examination of the dataset comprising 583 distinct meteorological recordings, several salient statistical features related to the 19 climatological variables emerged. Computation of central tendencies, particularly the mean and median values, paired with measures of dispersion such as standard deviation provide an in-depth overview of the data distribution and variability [18]. Among the studied variables, Tempmax, Tempmin, Temp, and Feelslikemax were of particular significance, with their respective statistics meticulously illustrated in Supplementary Table S2. The visualization in Supplementary Figure S2, which illustrates the chronological progression of the variables, serves as a vital tool for discerning temporal patterns, potential cyclical behaviors, or anomalies [19]. In light of the data presented, the correlation coefficients between DF cases and the variables Visibility, Solarradiation, Solarenergy, and Uvindex deserve particular attention. The observed Spearman’s rank correlation coefficients, although greater than 0.1, were still relatively modest in magnitude (Figure 2). Such coefficients suggest a potential link, albeit a weak one, between these meteorological parameters and the DF incidence. The connection between meteorological factors and vector-borne diseases, particularly dengue fever, has been a topic of interest in prior studies. For instance, Takuya Iwamura et al. [20] indicated that weather conditions could influence the life cycle of *Aedes* mosquitos, the primary vector for DF. In particular, elevated solar radiation, temperature, and UV index could either speed up the mosquito’s development or increase its breeding rates [21]. The delayed effect alluded to in our findings might be due to a time lag between the change in meteorological conditions and observable effects on the mosquito populations and, subsequently, dengue transmission [22]. Hence, while our correlation coefficients may seem low at first glance, they are consistent with the complex interplay of environmental and biological factors driving the dengue incidence.

In the realm of predictive modeling for DF in Singapore, the integration of meteorological determinants and their lag effects has proven to be pivotal. The delayed repercussions of these meteorological factors and the inherent cyclical trends of dengue propagation informed our decision to embed lagged values spanning from 1 to 12 weeks for the 19 meteorological variables, leading to the development of four diverse modeling approaches. The strategies ranged from a comprehensive incorporation of both lag and temporal factors to a model that bypassed both these elements. Mode 1, which melds both lag effects and temporal aspects, offers a comprehensive perspective on dengue forecasting. The scatter plot representation in Supplementary Figure S3 of observed versus predicted cases provides a visual overview of the model’s accuracy using several different machine learning algorithms, namely GLM, SVM, GBM, DT, RF, and XGBoost. Among these algorithms, the XGBoost model emerged as the most proficient, particularly in Modes 1, 3, and 4, as substantiated by its close alignment with observed cases. Intriguingly, in Mode 2, which solely focused on lag effects, SVM was the best performer, underscoring the dynamic nature of dengue prediction and the nuanced influence of input parameters. Supplementary Figures S4–S6, corresponding to Modes 2, 3, and 4, further demonstrate the models’ performances across different configurations. The robust performance of the XGBoost model in three out of four modes aligns with its known efficiency in handling complex datasets and non-linear relationships, a feature often seen in epidemiological data [23]. Conversely, the superior performance of SVM in Mode 2 might be attributed to its capacity to discern patterns in high-dimensional spaces, making it particularly adept at capturing the nuances of lag effects.

Our detailed analysis of predictive modeling effectiveness clearly shows that the choice of model significantly influences the accuracy of DF predictions. We used rigorous metrics like MAE, RMSE, and R-squared to examine the six different models. Interestingly, while many performance indicators showed similar results across models, the R-squared values exhibited distinct variations. These differences highlight the various capabilities of these models in explaining the variations in recorded dengue cases based on the provided predictors. Notably, the XGBoost model in Mode 4 stood out with an impressive R-squared value of 0.83, surpassing the other models. It demonstrated both minimal prediction errors and a significant ability to account for the variance in observed cases [24]. 

In the endeavor to elucidate the intricacies of our predictive models, we zeroed in on the XGBoost model under Mode 1 for a comprehensive dissection, given its commendable performance in the prior evaluations. By normalizing the relative significance of each meteorological determinant to a ceiling of 1, we obtained a quantitative measure of their influence, where higher scores are emblematic of augmented impact [25]. Our analysis identified the top 10 variables that predominantly affected the model’s predictions. Remarkably, the variable ‘Week’ emerged as a dominant factor with a score of 0.54, followed by Cloudcoverlag1, Cloudcoverlag5, and Preciplag5. Probing into the intricate web of associations between the predictors and dengue occurrence revealed compelling insights. Drawing from the significance scores derived from the XGBoost algorithm, 19 cardinal predictors manifested discernible affiliations with the dengue incidence, as illustrated in Supplementary Figure S7. Nonetheless, these affiliations were far from monolithic; rather, they exhibited non-linear correlations, as portrayed in Figure 3. The temporal dynamics of DF further amplify its complexity. Noteworthy is the cyclical pattern observed in its epidemiology, with discernible peaks and troughs over the past decade [26].

The nexus between cloud cover lag values for specific weeks and the subsequent decline in dengue cases illustrates the complex, multifaceted relationship between atmospheric conditions and disease propagation. Indeed, cloud cover can modulate the local microclimate, potentially affecting the lifecycle and breeding behavior of the *Aedes aegypti* mosquito, the primary vector for dengue transmission [27]. The surge in dengue cases observed with a precipitation lag for the fifth week exceeding 10 is noteworthy. Rainfall, especially when it accumulates in small, stagnant pools, provides ideal breeding grounds for the *Aedes* mosquito [28]. This relationship underscores the importance of considering not just the immediate effects of rainfall but also its lagged impact on dengue transmission, particularly in urban settings where water accumulation is common. Moreover, the rise in dengue cases corresponding with Dewlag7 is intriguing. Dew point temperature, a measure of atmospheric moisture, can influence mosquito behavior and survival. Elevated dew point temperatures might facilitate longer mosquito lifespans and enhance their ability to transmit the dengue virus [29]. The significance of meteorological predictors in determining DF transmission patterns is increasingly apparent, a notion corroborated by numerous epidemiological studies [30].

The application of machine learning in vector-borne disease epidemic risk prediction holds immense promise. Machine learning algorithms have the capacity to leverage vast datasets comprising environmental, epidemiological, and entomological variables, allowing for the development of robust predictive models [6]. These models can capture complex non-linear relationships and interactions among various factors that influence vector-borne disease transmission dynamics. Recent studies have exemplified the effectiveness of machine learning in studying vector-borne diseases like malaria [31], Zika virus infections [32], and Chagas disease [33]. Nonetheless, challenges remain in data quality, interpretability, and the need for continuous model validation. As machine learning methodologies continue to advance and data availability improves, they are poised to play a pivotal role in informing public health strategies, resource allocation, and outbreak preparedness in the context of vector-borne disease epidemics.

## 5. Conclusions

The last decade has showcased a tumultuous landscape of DF in Singapore, underpinning the multifarious factors at play in the transmission dynamics of this vector-borne ailment. Notably, meteorological determinants have emerged as pivotal contributors, highlighting the quintessential role of environmental parameters such as cloud cover, rainfall, and dew point temperatures in shaping the epidemiological trajectory of dengue. This research underscores the profound interplay between meteorological predictors and their lagged effects in determining the incidence rates, with distinct patterns elucidated through the adept XGBoost model. Among the predictors, temporal aspects, such as specific ‘Week’ and meteorological parameters like Cloudcoverlag1, Cloudcoverlag5, and Preciplag5, showcased significant influences. The non-linear associations between these predictors and the dengue incidence, vividly portrayed in our analyses, reiterate the complexity inherent in dengue’s transmission dynamics. Drawing from extensive data analytics and leveraging state-of-the-art machine learning algorithms, our study offers a comprehensive insight into potential predictive models for dengue fever. Furthermore, our findings accentuate the indispensable role of astute model selection, meticulous evaluation, and the synthesis of diverse determinants in crafting robust predictions. This confluence of data-driven insights and epidemiological understanding provides a foundation upon which targeted public health interventions can be structured, optimizing strategies for dengue prevention and control in Singapore and potentially other similar settings. Our research, echoing findings from seminal works in the realm of dengue research, reinforces the imperative to continually refine our understanding and leverage evolving data and technologies to fortify our defenses against such pervasive public health challenges.

## Figures and Tables

**Table 1:** Comparison of optimal model performance across different modes.

Metrics	Mode 1	Mode 2	Mode 3	Mode 4
	XGBoost	SVM	XGBoost	XGBoost
MAE	89.12	160.73	160.65	175.49
RMSE	156.07	268.83	232.58	247.86
$R^{2}$	0.83	0.5	0.49	0.42

Mode 1: incorporating both lag effects and temporal factors; Mode 2: considering only the lag effects; Mode 3: focusing solely on temporal factors; Mode 4: neglecting both lag effects and temporal factors. Mean Absolute Error = MAE, Root Mean Square Error = RMSE, and the coefficient of determination R-squared = $R^{2}$.

**Table 2:** Importance scores of top 10 variables from the XGBoost model in Mode 1.

Sequence	Feature	Importance	Cover	Frequency
1	Week	0.54	0.08	0.04
2	Cloudcoverlag1	0.10	0.01	0.01
3	Cloudcoverlag5	0.07	0.01	0.01
4	Preciplag5	0.03	0.01	0.01
5	Cloudcover	0.02	0.00	0.01
6	Dewlag7	0.02	0.00	0.00
7	Tempmax	0.02	0.01	0.08
8	Cloudcoverlag7	0.01	0.00	0.00
9	Cloudcoverlag3	0.01	0.00	0.01
10	Dewlag3	0.01	0.01	0.01

## Data Availability

Data available in a publicly accessible repository. The data presented in this study are available in the public data website (https://data.gov.sg/) and the weather data service website (https://www.visualcrossing.com/).

## Supplementary Materials

The following supporting information can be downloaded at: https://www.mdpi.com/article/10.3390/tropicalmed9040072/s1, Figure S1: The flowchart of the machine learning method; Figure S2: Time series plots of weekly climate variables from January 2012 to December 2022; Figure S3: (A). Observation and prediction of dengue cases in training data and test data of different models in Mode 1; (B). Timeline of observation and prediction of dengue cases in training data and test data of different models in Mode 1; Figure S4: (A). Observation and prediction of dengue cases in training data and test data of different models in Mode 2; (B). Timeline of observation and prediction of dengue cases in training data and test data of different models in Mode 2; Figure S5: (A). Observation and prediction of dengue cases in training data and test data of different models in Mode 3; (B). Timeline of observation and prediction of dengue cases in training data and test data of different models in Mode 3; Figure S6: (A). Observation and prediction of dengue cases in training data and test data of different models in Mode 4; (B). Timeline of observation and prediction of dengue cases in training data and test data of different models in Mode 4; Figure S7: The 18 most influential variables from the XGBoost model in Mode 1; Table S1: Number of dengue fever infections and deaths in Singapore from 2012 to 2022; Table S2: Quantitative characterization of weekly climatological variables; Table S3: Spearman correlation coefficient among variables; Table S4: Evaluation of the predictive efficacy among various models in Mode 1; Table S5: Evaluation of the predictive efficacy among various models in Mode 2; Table S6: Evaluation of the predictive efficacy among various models in Mode 3; Table S7: Evaluation of the predictive efficacy among various models in Mode 4.
