# Supplementary material for: Precision Prediction for Dengue Fever in Singapore: A Machine Learning Approach Incorporating Meteorological Data
Source: Trop Med Infect Dis. 2024 Mar 29;9(4):72. doi: 10.3390/tropicalmed9040072 (PMC11055163; doi:10.3390/tropicalmed9040072)
Supplement: Supplementary file 1 [file tropicalmed-09-00072-s001.zip › tropicalmed-2888115-supplementary.pdf]

Tropical Medicine and Infectious Disease

Supplementary Materials of Precision Prediction for Dengue Fever in Singapore: A Machine Learning Approach Incorporating Meteorological Data

Table S1. Number of dengue fever infections and deaths in Singapore from 2012 to 2022.

Year	Case	Death	Fatality Rate (‰)	χ^2	*p*-Value
2012	4632	2	0.43		
2013	22194	7	0.32		
2014	18326	5	0.27		
2015	11294	2	0.18		
2016	13085	11	0.84		
2017	2767	0	0.00	32.62	**0.000**
2018	3283	5	1.52		
2019	15998	21	1.31		
2020	35315	31	0.88		
2021	5264	5	0.95		
2022	32175	19	0.59		
Total	164333	108	0.66		

Table S2. Quantitative characterization of weekly climatological variables.

Metrics	Mean	Median	Mode	Standard Deviation	Variance	Q1	Q3
Sealevelpressure	1010.03	1010.00	1010.23	1.25	1.56	1009.24	1010.82
Solarradiation	172.74	167.97	187.46	42.72	1825.36	145.57	196.26
Winddir	140.33	156.74	25.34	70.61	4985.64	75.11	192.36
Cloudcover	79.44	84.36	84.79	9.80	95.94	68.68	85.93
Humidity	78.36	78.30	78.14	3.98	15.82	75.64	81.04
Precipitation	60.45	57.14	71.43	28.21	795.72	42.86	85.71
Feelslikemax	37.07	37.20	36.99	1.82	3.32	36.09	38.17
Tempmax	31.79	31.89	31.90	1.09	1.19	31.25	32.50
Feelslike	31.52	31.46	31.80	2.05	4.21	30.04	33.18
Temperature	28.26	28.27	28.27	0.92	0.84	27.67	28.91
Feelslikemin	26.56	25.96	25.99	1.96	3.84	25.11	27.80
Tempmin	25.72	25.64	26.59	0.88	0.78	25.09	26.31
Dew	23.93	24.00	24.09	0.69	0.48	23.63	24.33
Windspeed	17.47	16.93	16.33	3.69	13.60	15.11	19.12
Solarenergy	14.92	14.53	13.29	3.68	13.56	12.60	16.95
Visibility	9.61	9.76	9.93	0.77	0.59	9.49	9.97
Uvindex	6.37	6.29	5.71	1.56	2.44	5.21	7.57
Precipcover	5.81	5.36	4.17	4.47	20.00	2.98	7.74
Precipprob	5.32	4.14	0.00	5.73	32.79	1.39	7.35
Case	284.93	209.00	51.00	294.22	86566.48	81.00	372.50

Table S3. Spearman correlation coefficient among variables.

Metrics	Uvindex	Solarenergy	Solarradiation	Visibility	Cloudcover	Sealevelpressure	Winddir	Windspeed	Precipcover	Precipprob	Precipitation	Humidity	Dew	Feelslike	Feelslikemin	Feelslikemax	Temperature	Tempmin	Tempmax	Case
Humidity	-0.42	-0.49	-0.49	-0.38	-0.15	-0.19	0.06	-0.38	0.69	0.63	0.65	1.00	0.34	-0.52	-0.52	-0.24	-0.67	-0.53	-0.56	-0.12
Precipitation	-0.35	-0.42	-0.42	-0.38	-0.12	-0.22	0.26	-0.34	0.73	0.64	1.00	0.65	0.21	-0.31	-0.35	-0.15	-0.40	-0.40	-0.36	-0.08
Winddir	-0.35	-0.33	-0.33	-0.14	0.08	-0.35	1.00	-0.47	0.21	0.25	0.26	0.06	0.17	0.20	0.13	0.06	0.15	0.07	-0.06	0.02
Precipprob	-0.29	-0.41	-0.41	-0.34	-0.32	-0.27	0.25	-0.43	0.87	1.00	0.64	0.63	0.30	-0.23	-0.24	-0.07	-0.34	-0.27	-0.32	-0.12
Dew	-0.28	-0.33	-0.33	-0.14	-0.19	-0.28	0.17	-0.42	0.20	0.30	0.21	0.34	1.00	0.55	0.48	0.67	0.39	0.45	0.27	-0.07
Precipcover	-0.28	-0.39	-0.39	-0.33	-0.30	-0.25	0.21	-0.33	1.00	0.87	0.73	0.69	0.20	-0.36	-0.35	-0.22	-0.47	-0.38	-0.47	-0.09
Cloudcover	-0.11	0.00	0.00	0.04	1.00	-0.01	0.08	0.11	-0.30	-0.32	-0.12	-0.15	-0.19	-0.07	-0.11	-0.13	-0.02	-0.12	0.01	0.03
Feelslikemax	0.06	0.06	0.06	0.04	-0.13	-0.16	0.06	-0.23	-0.22	-0.07	-0.15	-0.24	0.67	0.82	0.69	1.00	0.78	0.68	0.85	-0.02
Feelslike	0.07	0.09	0.09	0.16	-0.07	-0.14	0.20	-0.10	-0.36	-0.23	-0.31	-0.52	0.55	1.00	0.93	0.82	0.97	0.91	0.73	0.06
Feelslikemin	0.08	0.08	0.08	0.19	-0.11	-0.08	0.13	0.00	-0.35	-0.24	-0.35	-0.52	0.48	0.93	1.00	0.69	0.91	0.98	0.61	0.06
Tempmin	0.11	0.11	0.11	0.21	-0.12	-0.04	0.07	0.02	-0.38	-0.27	-0.40	-0.53	0.45	0.91	0.98	0.68	0.90	1.00	0.62	0.07
Sealevelpressure	0.12	0.15	0.15	-0.05	-0.01	1.00	-0.35	0.40	-0.25	-0.27	-0.22	-0.19	-0.28	-0.14	-0.08	-0.16	-0.08	-0.04	-0.03	0.02
Temperature	0.16	0.19	0.19	0.21	-0.02	-0.08	0.15	-0.01	-0.47	-0.34	-0.40	-0.67	0.39	0.97	0.91	0.78	1.00	0.90	0.80	0.07
Visibility	0.20	0.20	0.20	1.00	0.04	-0.05	-0.14	0.25	-0.33	-0.34	-0.38	-0.38	-0.14	0.16	0.19	0.04	0.21	0.21	0.14	-0.08
Tempmax	0.25	0.30	0.30	0.14	0.01	-0.03	-0.06	-0.02	-0.47	-0.32	-0.36	-0.56	0.27	0.73	0.61	0.85	0.80	0.62	1.00	0.02
Windspeed	0.27	0.30	0.30	0.25	0.11	0.40	-0.47	1.00	-0.33	-0.43	-0.34	-0.38	-0.42	-0.10	0.00	-0.23	-0.01	0.02	-0.02	0.07
Solarradiation	0.87	1.00	1.00	0.20	0.00	0.15	-0.33	0.30	-0.39	-0.41	-0.42	-0.49	-0.33	0.09	0.08	0.06	0.19	0.11	0.30	0.22
Solarenergy	0.87	1.00	1.00	0.20	0.00	0.15	-0.33	0.30	-0.39	-0.41	-0.42	-0.49	-0.33	0.09	0.08	0.06	0.19	0.11	0.30	0.22
Uvindex	1.00	0.87	0.87	0.20	-0.11	0.12	-0.35	0.27	-0.28	-0.29	-0.35	-0.42	-0.28	0.07	0.08	0.06	0.16	0.11	0.25	0.21
Case	0.21	0.22	0.22	-0.08	0.03	0.02	0.02	0.07	-0.09	-0.12	-0.08	-0.12	-0.07	0.06	0.06	-0.02	0.07	0.07	0.02	1.00

Table S4. Evaluation of the predictive efficacy among various models in Mode 1.

Model	MAE	RMSE	R-squared
GLM	258.19	328.40	0.16
SVM	159.88	267.86	0.51
GBM	136.08	204.67	0.65
DT	158.85	260.59	0.44
RF	154.97	228.24	0.64
XGBoost	89.12	156.07	0.83

MAE = Mean Absolute Error; RMSE = Root mean square error; R-squared = R^2

Table S5. Evaluation of the predictive efficacy among various models in Mode 2.

Model	MAE	RMSE	R-squared
GLM	255.32	325.75	0.16
SVM	160.73	268.83	0.50
GBM	204.82	285.22	0.26
DT	233.76	366.36	0.00
RF	194.98	284.69	0.27
XGBoost	199.21	300.34	0.18

Table S6. Evaluation of the predictive efficacy among various models in Mode 3.

Model	MAE	RMSE	R-squared
GLM	203.90	293.14	0.20
SVM	170.09	288.99	0.27
GBM	179.66	252.08	0.40
DT	204.73	313.16	0.18
RF	172.87	260.45	0.42
XGBoost	160.65	232.58	0.49

Table S7. Evaluation of the predictive efficacy among various models in Mode 4.

Model	MAE	RMSE	R-squared
GLM	203.12	292.13	0.21
SVM	173.25	291.79	0.25
GBM	200.06	269.42	0.33
DT	196.08	304.75	0.20
RF	180.42	266.32	0.37
XGBoost	175.49	247.86	0.42

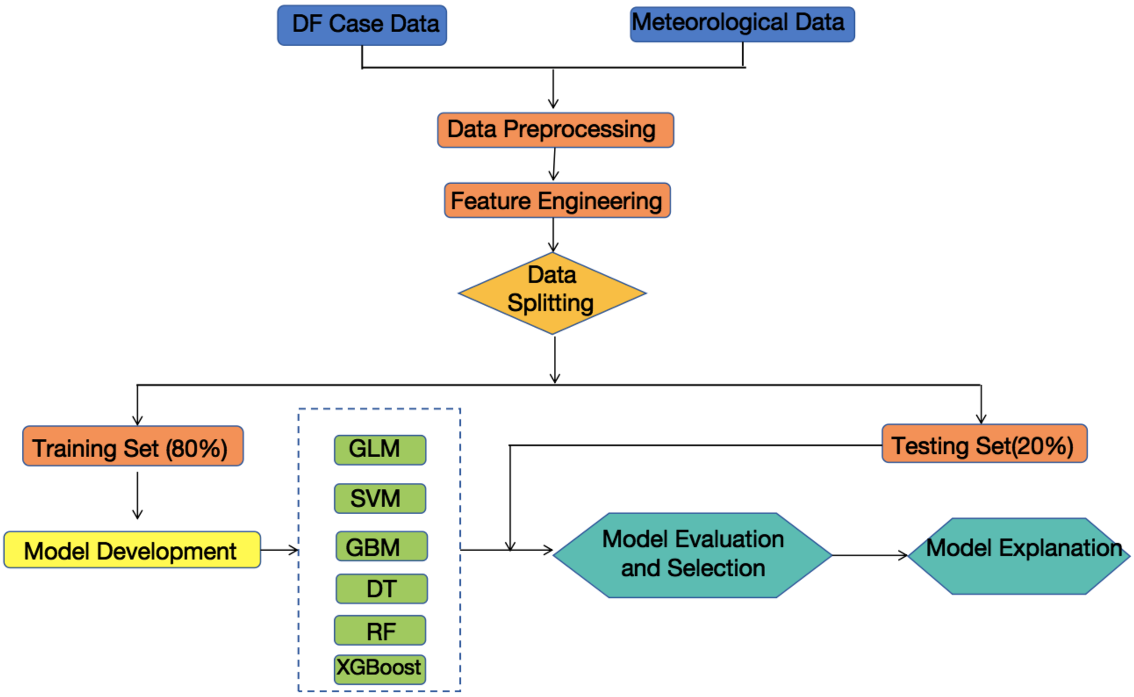

Figure S1. The flowchart of the machine learning method.

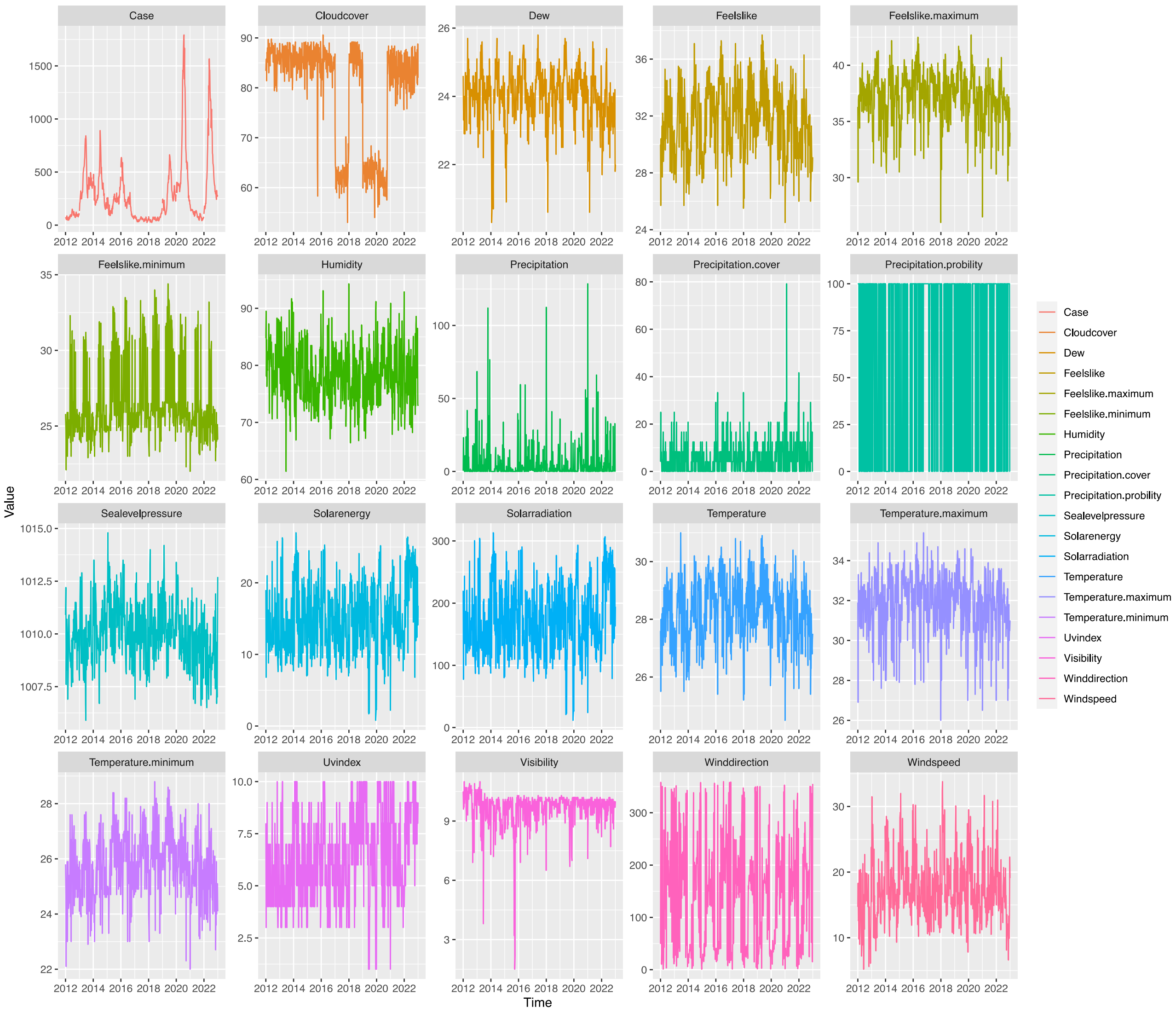

Figure S2. Time series plots of weekly climate variables from January 2012 to December 2022.

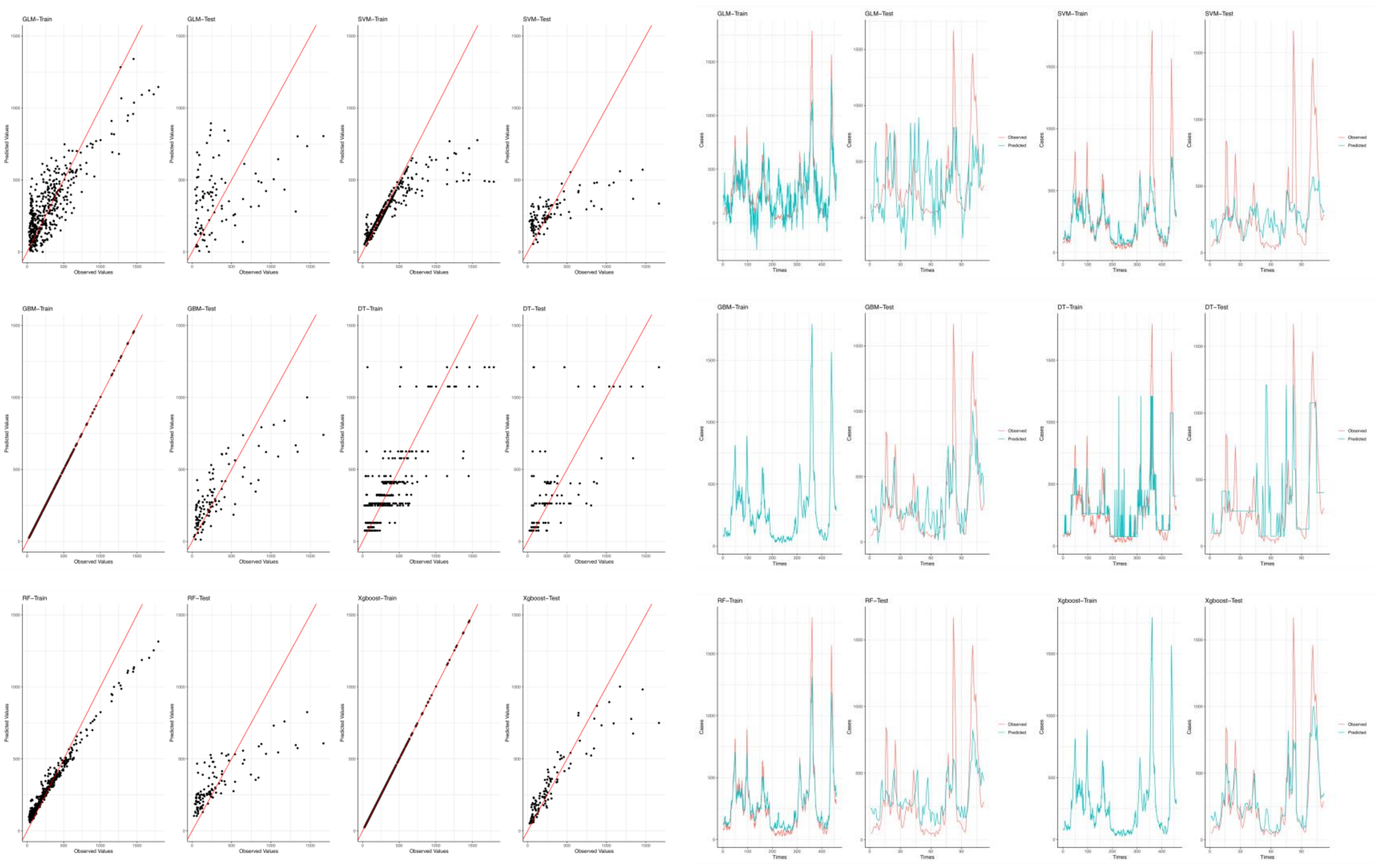

A B

Figure S3. (A). Observation and prediction of dengue cases in training data and test data of different models in Mode 1; **(B)**. Timeline of observation and prediction of dengue cases in training data and test data of different models in Mode 1 (orange means observation; blue means prediction).

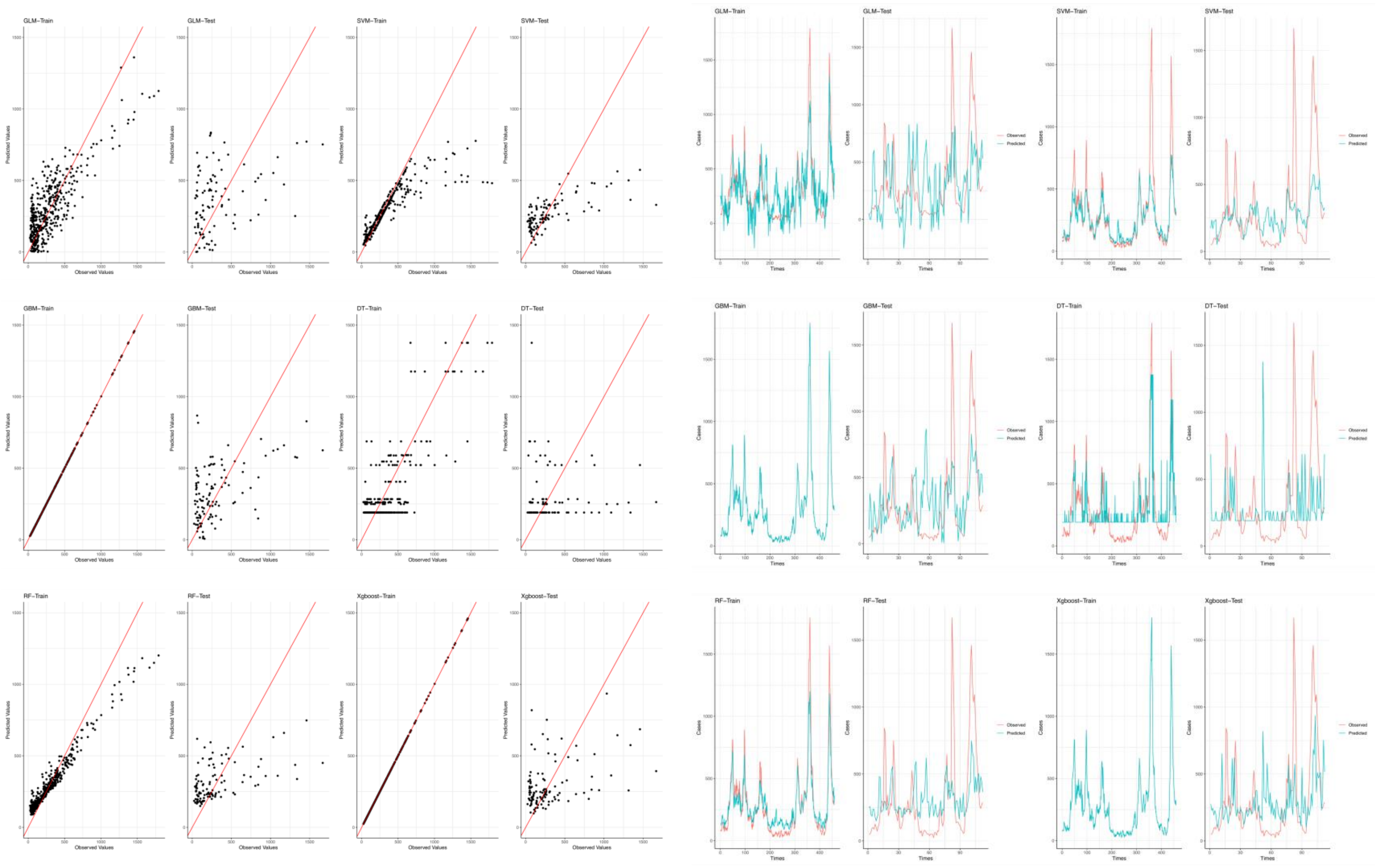

A B

Figure S4. (A). Observation and prediction of dengue cases in training data and test data of different models in Mode 2; **(B).** Timeline of observation and prediction of dengue cases in training data and test data of different models in Mode 2 (orange means observation; blue means prediction).

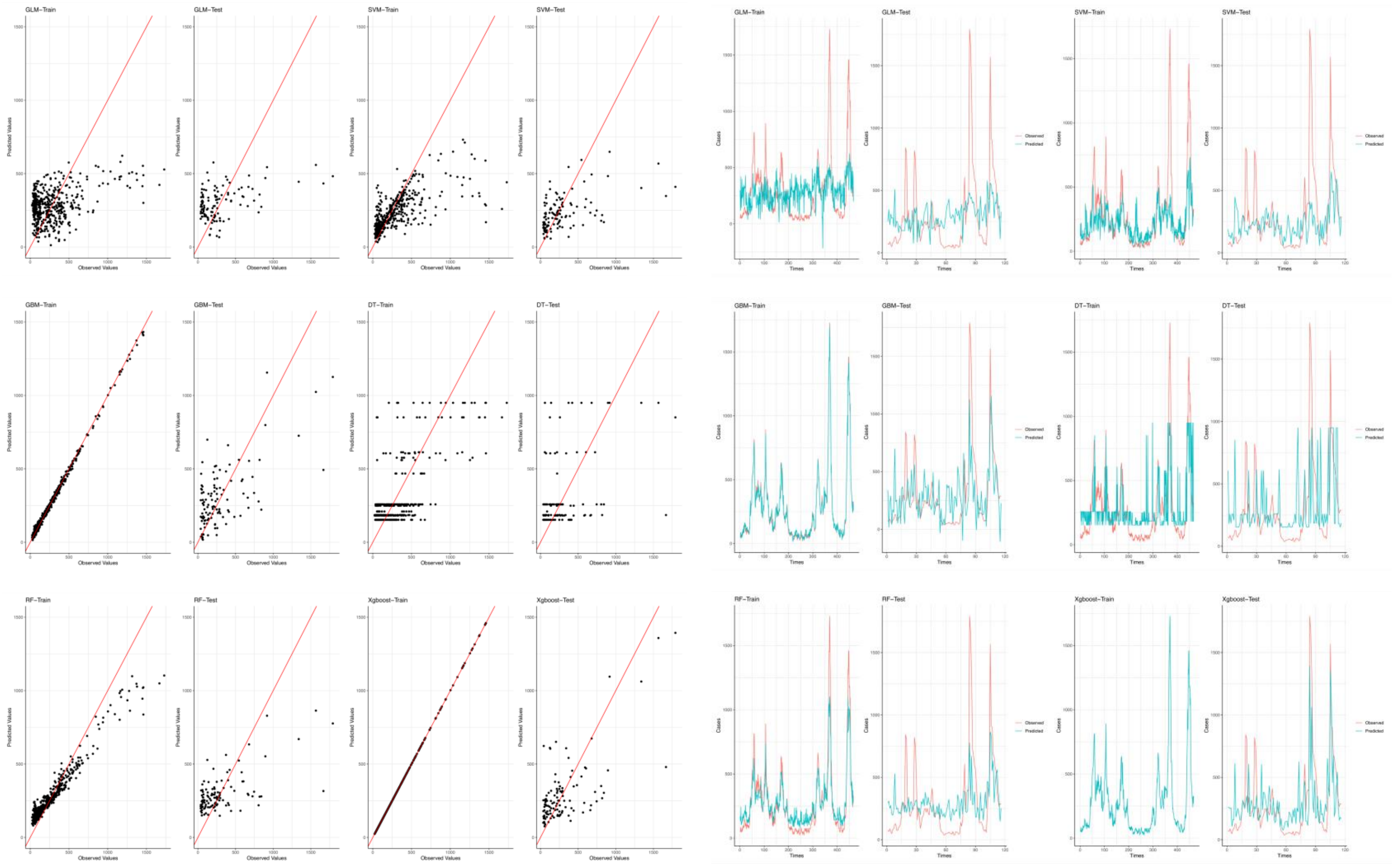

A B

Figure S5.(A). Observation and prediction of dengue cases in training data and test data of different models in Mode 3; **(B)**. Timeline of observation and prediction of dengue cases in training data and test data of different models in Mode 3 (orange means observation; blue means prediction).

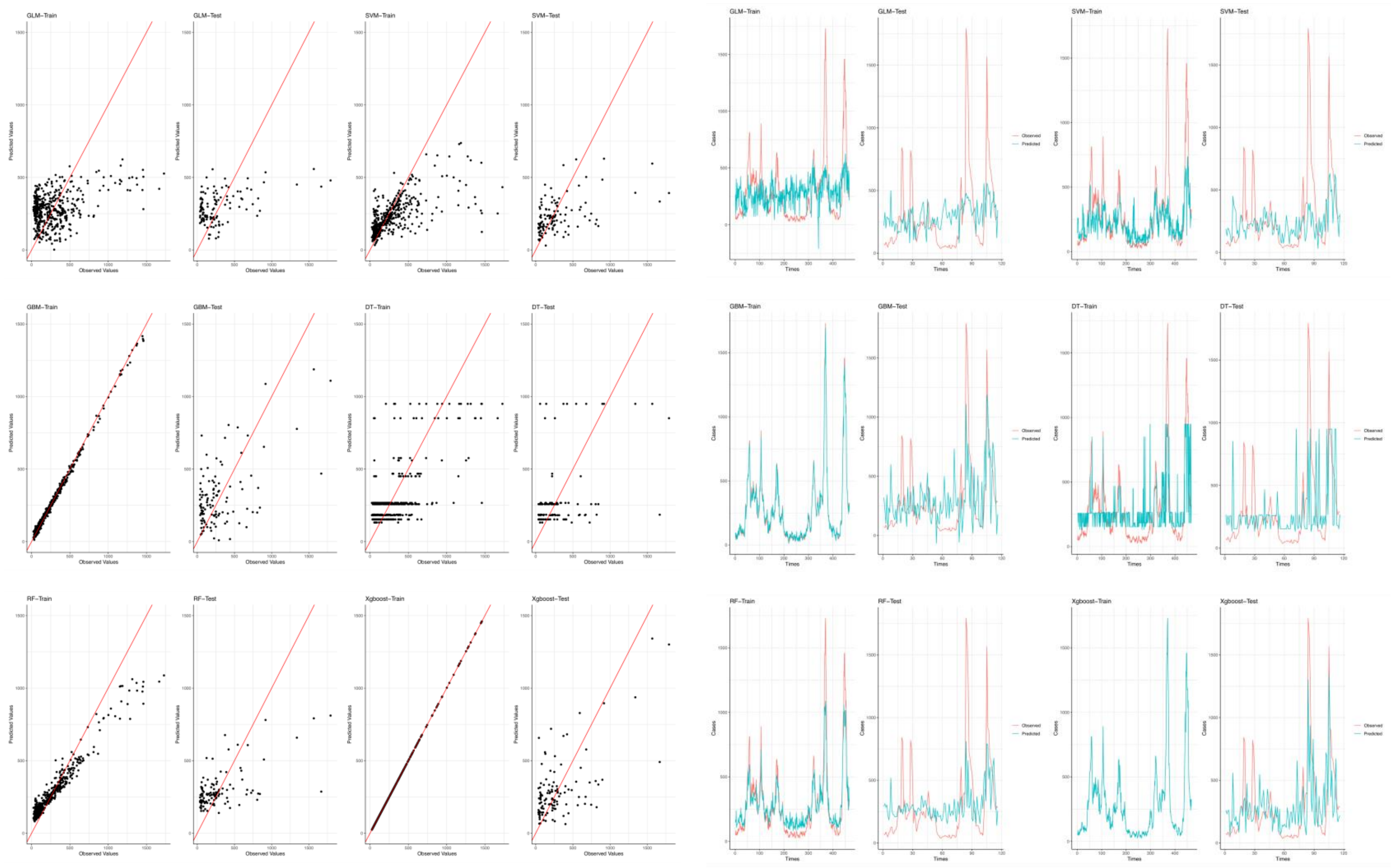

A B

Figure S6. (A). Observation and prediction of dengue cases in training data and test data of different models in Mode 4; **(B)**. Timeline of observation and prediction of dengue cases in training data and test data of different models in Mode 4 (orange means observation; blue means prediction).

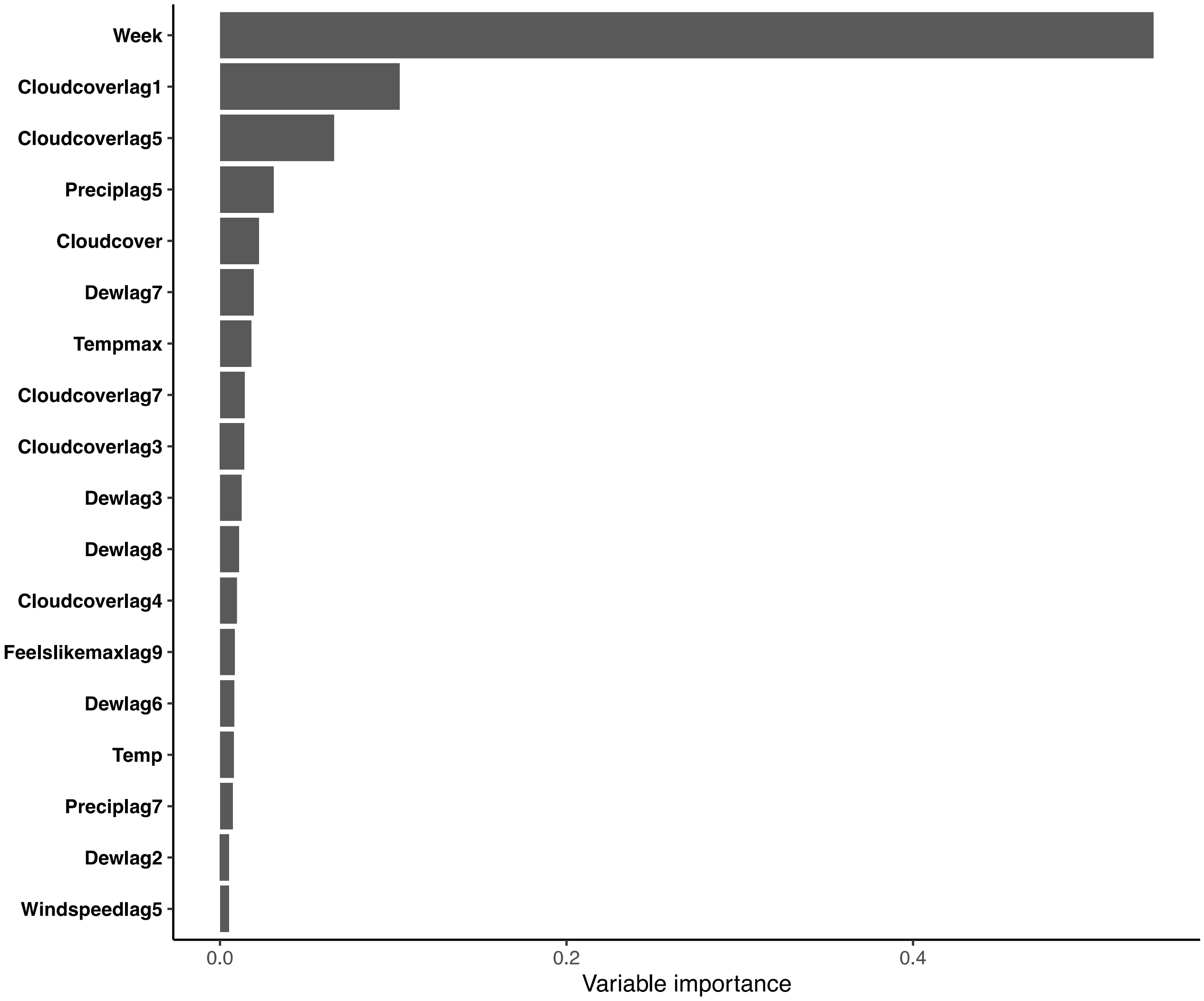

Figure S7. The 18 most influential variables from the XGBoost model in Mode 1.
